# Multi-Institutional Trends in Gynecological Robotic Surgery in India: A Real-World Scenario

**DOI:** 10.7759/cureus.36564

**Published:** 2023-03-23

**Authors:** Rooma Sinha, Vanita Jain, Somashekhar SP, Subhas C Saha, Chinnababu Sunkavalli, Lavanya Kiran, TS Shylasree, Kalyan Pandey, Girija S Mohanty

**Affiliations:** 1 Obstetrics and Gynecology, Apollo Health City, Hyderabad, IND; 2 Obstetrics and Gynecology, Post-Graduate Institute of Medical Education and Research, Chandigarh, IND; 3 Surgery, Manipal Hospital Bangalore, Bangalore, IND; 4 Surgery, Apollo Health City, Hyderabad, IND; 5 Obstetrics and Gynecology, Narayana Health Hospital, Bangalore, IND; 6 Obstetrics and Gynecology, Tata Memorial Hospital, Mumbai, IND; 7 Surgery, Manipal Hospital, Bangalore, IND

**Keywords:** minimally invasive surgery, india, trend analysis, robotic-assisted surgery, gynecological surgery

## Abstract

Background

Robot-assisted laparoscopic surgery in gynecology has grown exponentially compared to laparoscopic surgery. The probable reasons for the increased uptake of robotics are a shorter learning curve, three-dimensional vision, and increased dexterity compared to laparoscopic surgery, and precise surgery as compared to open surgery. This study compares the time trends of various parameters in robotic gynecological surgery in India over a decade.

Material and methods

In India, a retrospective analysis of all robot-assisted laparoscopic surgery for gynecologic diseases in five tertiary care hospitals was conducted between July 2011 and June 2021. Data were collected regarding demographic profiles, clinical and disease characteristics, and indications for surgery. Details related to surgery were collected, such as the number of ports, console and docking time, the procedure performed, total operative time, average blood loss, blood transfusion, and length of hospital stay. All the parameters collected were grouped into five years, and a comparison was made between the first five years (2011-2015) and the second five years (2016-2021). Statistical analysis, including descriptive statistics and trend analysis, was performed.

Results

During the 10 years, the total number of cases included was 1,501, out of which 764 were benign cases and 737 were pre-malignant/malignant cases. The common indications were uterine leiomyoma (31.2%) and carcinoma endometrium (28%). The mean age for benign cases was significantly lower than that for malignant cases (40.84 years and 55.42 years, respectively). Mean blood loss was significantly lower for benign indications (97.48 mL) than for oncological surgery (184.67 mL) and needed fewer transfusions. The mean length of stay (LOS) for benign (2.07 days) and malignant/ pre-malignant cases (2.32 days) and the mean BMI for benign (28.40) and for oncological patients (28.47) were similar in both groups. The docking time reduced significantly in the last five years.

Conclusion

The current retrospective study demonstrates an increasing uptake of robotic technology in gynecological surgery in India. Of the total cohort of cases, 70.9% of patients underwent gynecological robotic surgery in the last five years. A burst of adaptability happened for malignant cases in 2017 and benign cases in 2018, probably due to the increased availability of robotic platforms and improved awareness of technology and training among medical professionals. The number of cases has grown exponentially over the last five years in both benign and malignant/ pre-malignant scenarios; however, there has been a downward trend in the robotic surgery performed in the previous couple of years due to the uncertainty of the COVID pandemic.

## Introduction

The advances in surgery have been phenomenal in the past 50 years, with a paradigm shift from open to laparoscopic surgery. Due to ease of access, the trend is rapidly changing toward robotic surgery all around the globe. This has been driven by the safety of curiosity, better facilities, and the hunger to improve. Minimally invasive surgery (MIS) reduces perioperative morbidity and improves the quality of life. Robot-assisted laparoscopic surgery (RALS) provides all the benefits of keyhole surgery, such as three-dimensional magnified panoramic vision, tremor-free, and intuitive movements. It is the logical advancement of laparoscopic surgery and complements it well. The FDA approved RALS for gynecological diseases in 2005 [1]. In India, the present-day robots made their way around 2011. Due to factors such as increased awareness among patients, availability of more trained surgeons, and coverage by insurance, the demand for robotic surgery is on the rise. The current study aims to share the trend of RALS for benign and malignant/ pre-malignant gynecological conditions over the last decade at five tertiary care centers across India. We aim to analyze the changing trend of robotic surgery in gynecology in the previous 10 years and discuss adaptations made by surgeons to make robotic surgery affordable and feasible in the Indian scenario. This retrospective analysis of prospectively collected data will help to give insight into trends in the last 10 years and predict future trends in gynecologic robotic surgery in India.

## Materials and methods

The data were collected from five (out of eight centers invited for study) tertiary care robotic centers across India (Apollo Health City, Hyderabad; Postgraduate Institute of Medical Education and Research [PGIMER], Chandigarh; Manipal Hospital, Bangalore; Narayana Hrudayalaya Hospital, Bangalore; and Tata Memorial Hospital [TMH], Mumbai). These centers voluntarily provided data as per the fixed data collection sheet shared with each center. Each center provided data on all patients who underwent gynecologic robotic surgery between July 2011 and June 2021. The data were entered into an appropriate spreadsheet. The data were prospectively collected, cleaned, coded, exported to the SPSS software (IBM Corp., Armonk, NY), and analyzed retrospectively. This study aimed to evaluate the changing scenario and trends of robotic surgery in India over the years of introducing robotic platforms in India. The total study duration was divided into two groups. The initial and last five years of study results were compared. All centers in this study used the da Vinci platform, either Si or Xi, and the surgery was performed by a robotically trained specialist in each medical center. Demographic data of age and body mass index (BMI) were recorded. Robotic surgeries with multiple ports included those with additional/ reduction of robotic arm use with an additional laparoscopic port for a surgical instrument manipulated by an assistant. Diagnoses were categorized as benign or malignant/pre-malignant diseases and then subcategorized according to the histopathological finding. Benign diseases included uterine leiomyoma, adenomyosis, pelvic endometriosis, pelvic organ prolapse, chronic tubal ectopic pregnancy, dermoid cyst, endometriotic cyst, and vault prolapse. Malignant and pre-malignant diseases include endometrial cancer, cervical cancer, ovarian cancer, cervical intraepithelial neoplasia II/III, and endometrial hyperplasia.

Statistical analysis

The continuous data were reported with mean and standard deviation. The categorical data were reported with frequencies and percentages. Bar charts were used for the graphical display of categorical and continuous data. SPSS version 26.0 for Windows was used to analyze the collected data. The Spearman correlation test was conducted to analyze the relationship between categorical variables. Spearman correlation analysis was performed, with ρ indicating the crude Spearman coefficient. A p-value of less than 0.05 was considered statistically significant.

## Results

**Figure 1:**
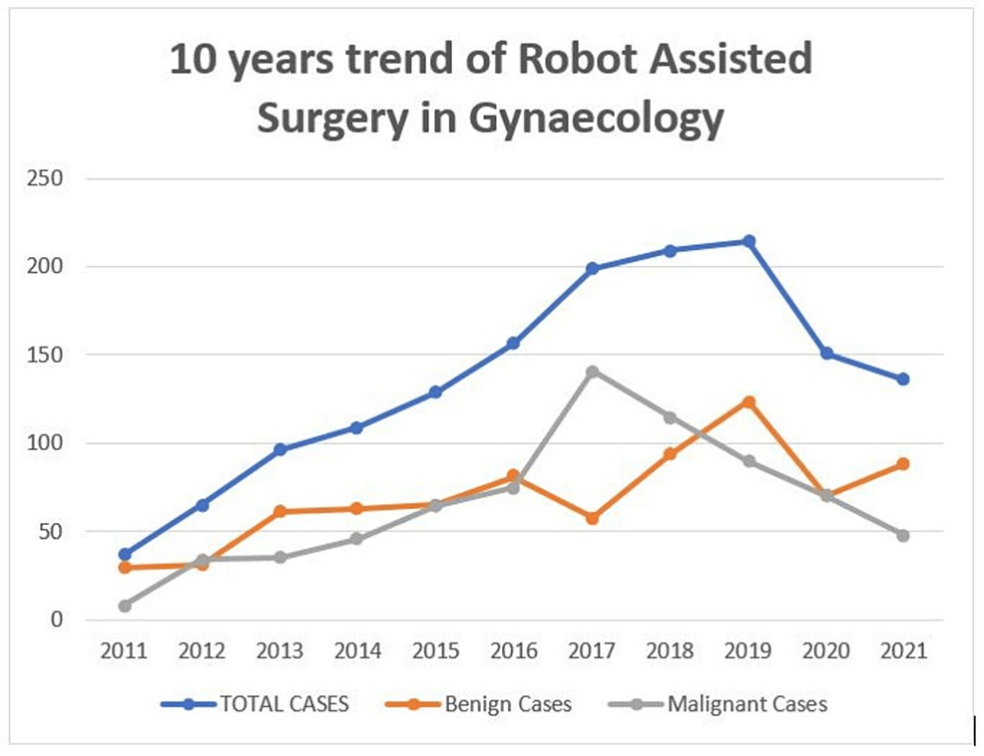
Trend shows the year-wise distribution of robot-assisted laparoscopy surgery for both benign and malignant/ pre-malignant cases in gynecology. Blue line indicates trend of total gynecological cases that underwent robot-assisted laparoscopic surgery. Orange line indicates trend of benign gynecological cases that underwent robot-assisted laparoscopic surgery. Gray line indicates trend of malignant gynecological cases that underwent robot-assisted laparoscopic surgery.

The total number of cases operated by RALS for gynecological etiology between 2011 and 2021 was 1,501, out of which 764 were benign cases and 737 were malignant and pre-malignant cases. The highest number of cases (214) was operated in 2019 (Figure 1).

**Figure 2:**
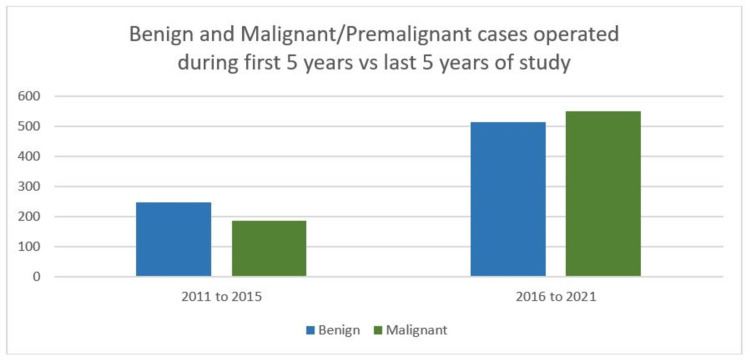
Bar diagram showing benign and malignant/pre-malignant cases operated on during the first five years versus the last five years of study. Total benign gynecological cases operated by robotic platform (blue) in the first five years were 240 and that in the last five years were 510 cases. Total malignant gynecological cases operated by robotic platform (green) in the first five years were 192  and that in the last five years were 559 cases.

Statistical analysis shows that only 29.1% of cases underwent gynecological robotic surgery over the initial five years, compared to 70.9% of patients who underwent gynecological robotic surgery over the last five years of the current study, demonstrating the rapid acceptability of robots for gynecological procedures. Although robotic surgery was initially used for malignant conditions, there was increased adaptability for benign diseases after 2017 (Figure 1). Over the last decade, the proportion of malignant/ pre-malignant and benign cases operated by robotic assistants has been 49.1% and 50.9%, respectively. Statistical analysis showed that benign gynecological cases operated on from 2011 to 2015 versus 2016 to 2021 were 32.6% and 67.4%, respectively. Similarly, patients with gynecological malignancy/pre-malignant cases operated on from 2011 to 2015 versus 2016 to 2021 were 25.4% and 74.6%, respectively. There was a significant increase in the use of robotic surgery in both the malignant and benign cases when the first five years were compared to the last five years (p=0.002) (Figure 2).

The common benign cases for which robotic surgery was performed include uterine leiomyoma (31%), endometrioma (8%), pelvic endometriosis (6%), adenomyosis (4%), vault prolapse (3%), adnexal masses (1%), abnormal uterine bleeding (1%), and ectopic pregnancy (1%). Robot-assisted laparoscopic myomectomy contributed a maximum of 31% among benign diseases operated on over the study period. Common malignant and pre-malignant diseases were carcinoma endometrium (28%), endometrial hyperplasia (11%), carcinoma cervix (4%), carcinoma ovary (1%), and cervical intraepithelial neoplasia (CIN - II/III) (1%). Robot-assisted laparoscopic hysterectomy, bilateral salpingo-oophorectomy, and bilateral pelvic lymph node dissection for endometrial cancer contributed a maximum of 28% among malignant diseases operated over the study period. The mean age for benign cases was significantly lower than malignant cases, 41 years and 55 years, respectively. The mean BMI for benign and malignant patients was 28.4 and 28.4, respectively, and was not statistically different (p=0.760). Mean blood loss was significantly higher in oncological surgery (184.67 mL) when compared to surgeries performed for benign indications (97.48 mL). The requirement for blood transfusions for benign cases (0.02 units of packed red blood cells [PRBCs]) was significantly lower than for malignant cases (0.05 units of PRBCs). The mean length of stay (LOS) for benign cases (2.07 days) and oncological cases (2.32 days) and the mean BMI for benign cases (28.407) and oncological cases (28.476) were similar in both groups (Table 1).

**Table 1:** Comparison of age, BMI, blood loss, and blood transfusion for benign versus malignant/premalignant gynecological cases operated on by robot-assisted laparoscopic surgery during 2011 to 2021. BMI, body mass index

	Category	Number of cases operated	Mean	Standard deviation	Standard error mean	P-value
Age	Benign	764	40.84	10.515	0.380	<0.001
	Malignant	737	55.42	10.366	0.382	
BMI	Benign	764	28.407	4.5319	0.1640	0.760
	Malignant	737	28.476	4.2022	0.1548	
Blood loss	Benign	764	97.48	162.072	5.867	<0.001
	Malignant	737	184.67	157.972	5.819	
Blood transfusion	Benign	764	0.02	0.263	0.010	0.003
	Malignant	737	0.05	0.172	0.006	

**Figure 3:**
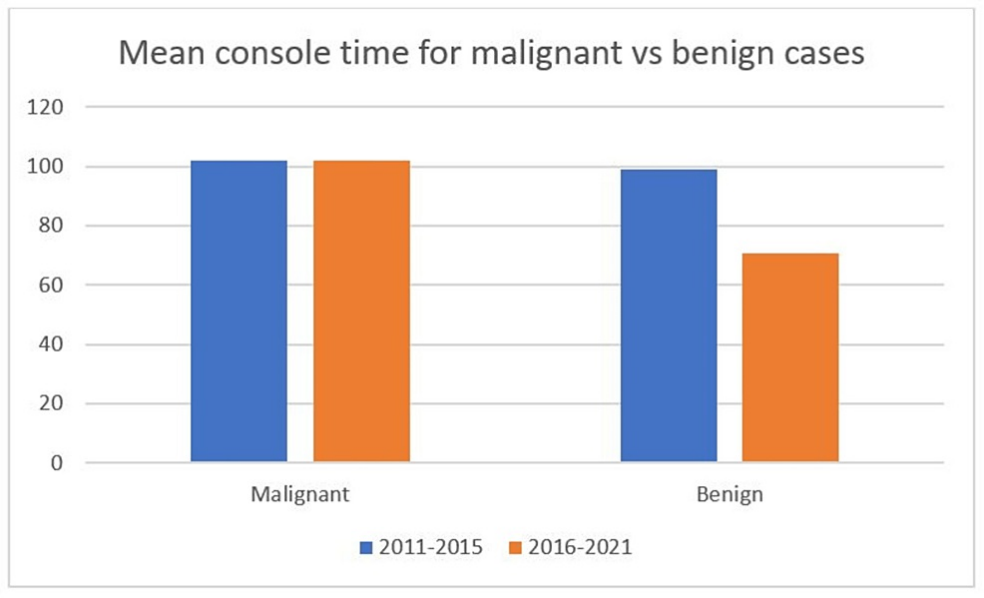
The bar diagram showing the comparison between the mean console time (in minutes) for malignant/pre-malignant and benign gynecological cases operated during 2011 to 2015 and 2016 to 2021.

When comparing oncological cases between the first five years and the last five years, we found that the mean console time did not change much, as more complex surgeries were performed during the last five years of study. The console time during 2011 to 2015 was 102.08 minutes, and that during 2016 to 2021 was 102.13 minutes. However, we analyzed the mean console time for benign patients and found that it was significantly reduced in the last five years (p=0.02). The mean console time for benign cases during 2011 to 2015 and 2016 to 2021 was 99.18 minutes and 70.56 minutes, respectively (Figure 3).

Similarly, the mean LOS during 2011 to 2015 and 2016 to 2021 was 2.12 days and 2.23 days, respectively. However, there has been a significant decrease in docking time during the last five years compared to the first five years of introducing robotic surgery in India (Table 2).

**Table 2:** Comparison of docking time, post-operative stay, and blood transfusion for gynecological cases operated on by robot-assisted laparoscopic surgery during 2011 to 2015 versus 2016 to 2021. PRBC, packed red blood cell

	Category	Number of cases operated	Mean	Standard deviation	Standard error mean	P-value
Docking time (minutes)	2011 to 2015	436	26.75	16.046	.768	<0.001
	2016 to 2021	1065	14.70	8.761	.269	
Length of stay (days)	2011 to 2015	436	2.12	.835	.040	0.208
	2016 to 2021	1065	2.23	1.733	.053	
Blood transfusion (units of PRBCs)	2011 to 2015	436	.05	.263	.013	0.202
	2016 to 2021	1065	.03	.205	.006	

The requirement for blood transfusion over the years remained the same. The overall minor complications in this study were port site discomfort/pain, minimal bleeding during surgery <2%, and major complications such as port site hernia, bowel injury/bowel ischemia/ gangrene, or massive bleeding due to vessel injury <1%. Robotic surgery in gynecology showed an increasing trend except for the last two years because of the COVID pandemic, as per the current study.

## Discussion

India had its first da Vinci system in 2002, which was used for cardiac and thoracic procedures. However, the first robot-assisted gynecologic procedure was performed in 2007. There is an increased adaptation of both diagnostic and therapeutic techniques in medical branches, including radiodiagnosis, radiotherapy, and surgical branches. The rate of adaptability for diagnostic tools is faster than therapeutic techniques. Gradually, over decades, laparoscopic surgeries were incorporated and are currently widely used for many surgical branches, leading to a decrease in conventional open surgeries. Since the introduction of the robotic platform for surgery, it has come a long way and is now standard practice in both benign and malignant gynecological surgery. The use of robotics provides several advantages. Most importantly, a stable binocular stereoscopic three-dimensional high-definition camera with 15x magnification vision and endo-wrist (7 degrees of freedom) intuitive instrument movements, which have the ability of tremor filtration and give increased dexterity and precision both in dissection and suturing. The ergonomic advantage for surgeons to sit and comfortably operate with arms rested reduces surgeon fatigue in long surgical procedures. Due to the aforementioned benefits of robotic surgery over laparoscopic surgery, the robotic platform is rapidly adopted among surgeons of various branches as another advance in medical technology. As per the intuitive surgical sustainability report 2021, globally, 6,700 da Vinci surgical systems were installed, and more than 10 million procedures were performed. In India, more than 90 da Vinci surgical systems were installed, and more than 50,000 procedures were performed till 2021 [2]. Performing laparoscopy is skill-dependent and complex, and has long learning curves. Early estimates of minimally access surgery cases required to gain proficiency are around 35-40 operations. However, approximately 25 cases are required for the competent performance of a retropelvic lymphadenectomy. The learning curve in robotic surgery is short and early proficiency compared to laparoscopic surgery [3]. This short learning curve and the availability of more than 90 systems across India are reflected in the increased adaptivity of this technology among Indian gynecological surgeons, as reported in the current study. In the United States, gynecology was next to urology among surgical specialties that used robotic surgery in 2021. The same is the case in India, with gynecology being second only to urology. Robotic surgeries have increased by 10-20% yearly worldwide, including in the current study, except during the COVID pandemic [4]. According to the current study, multiple gynecological surgeries were performed robotically, such as hysterectomy and bilateral salpingectomy, myomectomy/adenomyomectomy, endometriosis excision, and sacro-colpopexy. These surgeries, as per the American College of Obstetricians and Gynecologists (ACOG) Committee Opinion September 2020, can be performed safely using a robotic platform [5]. Patients scheduled for gynecologic procedures of short duration and low complexity, such as tubal ligation, simple ovarian cystectomy, and diagnostic laparoscopy, are unlikely to benefit from robot-assisted surgery [5]. The current study shows that complex robotic surgeries such as stage 3/4 endometriosis, complex leiomyoma, uterine size of 18-20 weeks for hysterectomy, and, in gynecology malignancy cases, para-aortic lymph node dissection were increased over the last five years. One of the advantages of using a robotic platform is reduced conversions (conversion to open surgery from minimally invasive robotic surgery) in complex gynecological diseases. In the current study, two conversions from robotic surgery to open surgery was done in view of large and multiple myoma uterus [6]. This was also during the first five years. Although the current study was not a comparative study, the historical laparoscopic data in the world show that the conversion rate is around 0-19% and up to 25% in gynecologic malignancy. This wide range of complications was due to different surgeons with different levels of skills. Still, across all centers included in the current study, the conversion rate of robotic surgery is below 1% in India [7-10]. The advantages of MIS are less pain, early postoperative recovery, lower incidence of incisional hernia, and better cosmesis, which has resulted in increased demand for laparoscopic and robotic surgery. As per the current study, robotic surgery showed slow adaptability during the initial few years, but a burst of adaptability happened for malignant cases in 2017 and benign cases in 2018. The demand for robotic platforms is rapidly increasing because of the short learning curve for surgeons, more awareness, and demand by patients. According to the current study, there was a statistically significant increase in both benign and malignant cases operated by robotic systems in gynecology. As per the current study, the mean age for benign and malignant/pre-malignant cases was 40.84 and 55.42, respectively, similar to the mean age reported by Yoo et al. [11]. The benefit of the robotic platforms in large and multiple fibroids is especially useful. In their paper, Lee et al reported that 68% of all benign cases were performed for uterine fibroids. In the current study, fibroid uterus topped the list in benign robotic surgery for both myomectomy and hysterectomy. Ease of manipulation of the large specimens (up to 20 weeks in size) with robotic arms gives the advantage in hysterectomy in such cases. Myomectomy being a suture-intensive surgery, intuitive, and wristed suturing technique gives an advantage and is the reason for more adaptability [12]. We report a significant increase in gynecologic robotic surgery in malignant cases. Sentinel lymph node biopsy for carcinoma endometrium with an integrated near-infrared camera and ICG (indocyanine green) has become a standard of care. The rise in the use of robotic platforms for oncological cases is explained by the increased confidence of surgeons to use robotic systems, which has reduced open surgery for similar indications. Pelvic and para-aortic lymphadenectomy can be adequately performed by a robot, leading to widespread acceptance. Inbuilt technology to do Sentinel node sampling where indicated can be performed much easier in a shorter time and with less blood loss with the use of a robotic platform [13]. A randomized controlled trial in Finland showed that the outcomes were similar between the laparoscopy and robotic groups for endometrial cancer, but robotic surgery cases reported improved patients' quality of life, with benefits noted up to 12 weeks after surgery [14]. Bernardini et al reported that robotic surgery would be a safe and effective option in the surgical treatment of endometrial cancer, especially in obese women [15-17]. Robotic surgery is easier to learn for surgeons with lesser experience but requires special training [18]. The benefits of robotic surgery are enhanced with da Vinci Xi surgical system's new, safer version. If we look at the trend in the current study, there was a gradual increase in robotic surgery performed for oncological cases till 2017, of which the leading indication was endometrial cancer. This rise was followed by a decreased trend, especially due to the conclusion of a reduced disease-free survival rate in minimally invasive surgery compared to open surgeries for cancer cervix patients in the Laparoscopic Approach to Cervical Cancer (LACC) trial [19]. The COVID pandemic was also responsible for decreased number of cases to a certain extent in the years 2020 and 2021. We must wait for the results of the ongoing trial in Europe, titled "Robotic-assisted Approach to Cervical Cancer (RACC) trial," which compares the oncologic outcomes of open surgery and robotic surgery in early cervical cancer [20]. The current study confirms the reduced LOS in cases who underwent robotic surgery, which translates to a reduction in the indirect cost of surgery [21]. There has been a significant decrease in docking time over the years. The requirement for blood transfusion remained low over the years. Some studies reported less blood loss and blood transfusion requirements after robotic surgeries [22,23]. The economy plays a major role in the adoption of new technology the world over and has special implications in a country like India [21]. The majority of the population in India pays either by government-sponsored health schemes or out of pocket. Both, at present, do not cover the robotic surgery cost. Private insurance currently covers robotic surgery costs, which are often limited. Reducing the disposable cost by adopting a two-arm robotic surgery technique and increasing the volume of cases can help improve accessibility for all patients [24,25]. Robotic surgery can lead to a reduced hospital stay, which can compensate for the extra surgery cost. Secondly, using the same instruments to perform multiple functions, such as using pro grasp forceps for both retraction and needle holders, reduces consumables costs. The overall minor complications such as port site discomfort/pain and minimal bleeding during surgery were <2%, and major complications such as port site hernia, bowel injury/bowel ischemia/gangrene, or massive bleeding due to vessel injury were <1% [26]. Acceptable low morbidity and complications were seen in the current study and have been similarly reported in the literature [27,28]. The limitations of the current study are as follows: in India, we have four different robotic platforms approved for clinical use, and under this study, all cases were operated by the da Vinci system, and not all centers in India performing RALS for gynecological cases have participated for the study.

## Conclusions

This data analysis is a real-world scenario from India. The current retrospective study demonstrates an increasing uptake of robotic technology in gynecological surgery in India, especially in the last five years. Of the total cohort of cases, 70.9 % of patients underwent gynecological robotic surgery in the last five years. A burst of adaptability happened for malignant cases in 2017 and benign cases in 2018, probably due to the increased availability of robotic platforms and improved awareness of technology and training among medical professionals. The number of cases has grown exponentially over the last five years in both benign and malignant/ pre-malignant scenarios; however, there has been a downward trend in the robotic surgery performed in the previous couple of years due to the uncertainty of the COVID pandemic. Translating all the advantages of MIS and reducing the need for open surgery for complex gynecological surgeries even by the surgeons in their early clinical practice will be the most attractive reason for them to adopt this technology. Hence, this rise in trend is only the tip of the iceberg, and it will not be wrong to predict an exponential rise in RALS for gynecological indications in the coming decade.
